# Laparoscopic Gastric Plication versus Laparoscopic Sleeve Gastrectomy: An Up-to-Date Systematic Review and Meta-Analysis

**DOI:** 10.1155/2018/3617458

**Published:** 2018-10-09

**Authors:** Konstantinos Perivoliotis, Eleni Sioka, Georgia Katsogridaki, Dimitrios Zacharoulis

**Affiliations:** Department of Surgery, University Hospital of Larissa, Mezourlo, 41110 Larissa, Greece

## Abstract

**Introduction:**

A meta-analysis was conducted in order to provide an up-to-date comparison of laparoscopic sleeve gastrectomy (LSG) and laparoscopic gastric plication (LGP) for morbid obesity.

**Materials and Methods:**

The PRISMA guidelines and *the Cochrane Handbook for Systematic Reviews of Interventions* were used for the conduction of this study. A systematic literature search was performed in the electronic databases (MEDLINE, CENTRAL, and Web of Science and Scopus). The fixed effects or random effects model was used according to the Cochran Q test.

**Results:**

Totally, 12 eligible studies were extracted. LSG displayed a statistically significant lower rate of overall complications (OR: 0.35; 95% CI: 0.17, 0.68; *p*=0.002) and a sustainable higher %EWL through all time endpoints (OR: 4.86, *p*=0.04; OR: 7.57, *p* < 0.00001; and OR: 13.74; *p* < 0.00001). There was no difference between the two techniques in terms of length of hospital stay (*p*=0.16), operative duration (*p*=0.81), reoperation rate (*p*=0.51), and cost (*p*=0.06).

**Conclusions:**

LSG was demonstrated to have a lower overall complications and a higher weight loss rate, when compared to LGP. Further RCTs of a higher methodological quality level, with a larger sample size, are required in order to validate these findings.

## 1. Introduction

### 1.1. Rationale

Obesity, defined as body mass index (BMI) ≥ 30 kg/m^2^, has become a worldwide epidemic, over the last decades, with an increasing incidence trend, not only in the Western world [1, 2] but also in the developing countries [3] as well. More specifically, the current overall obesity prevalence is 5% in children and 12% in adults [1], while according to recent estimations, in the next decades, almost 38% and 20% of the worldwide adult population will be classified as overweight and obese, respectively [2].

Besides the vast psychological [4, 5] and socioeconomic effects [4, 5] of this trending condition, obesity has been directly associated with various comorbidities, such as hypertension [6], type II diabetes [7], and sleep apnea [8] and increased risk for certain malignancies [8]. According to the literature, even a modest weight loss (5 to 10%) results in a respectable decrease in systolic pressure, elevation of apnea symptoms, metabolic normalization, and improvement in quality of life aspects, like mobility and sexual function [9].

As a consequence, tackling the emerging obesity issue has become a priority in the healthcare systems of many countries [1, 2]. Due to the fact that lifestyle intervention, dietary changes, and pharmacotherapy do not achieve an adequate long-term weight loss ratio, when compared to operative management [10, 11], the notion of bariatric surgery has emerged [12, 13]. Bariatric operations are classified, according to their gastrointestinal effect, in restrictive (e.g., adjustable gastric band, vertical banded gastroplasty, and sleeve gastrectomy), malabsorptive (e.g., jejunoileal bypass and biliopancreatic diversion with duodenal switch), or combined techniques (e.g., Roux-en-Y gastric bypass) [14]. Moreover, the advent of the minimal invasive era was also characterized by the introduction of the laparoscopic principles in bariatric surgery [15].

Laparoscopic sleeve gastrectomy (LSG) was designed as a part of a two-stage procedure for morbidly obese patients, but gradually evolved as a standalone operation [16]. As a result of the satisfactory results compared to the rest of the abovementioned procedures, the infiltration of laparoscopic sleeve gastrectomy among bariatric surgeons has increased over the past years [16, 17]. Staple line leak [18–20] and haemorrhage [21], though two major and difficult-to-treat postoperative complications, have been extensively investigated in current literature, mainly due to the related morbidity and mortality rates.

Subsequently, an alternative restrictive procedure, laparoscopic gastric plication (LGP), has been proposed by Tretbar et al. [22] in 1976 and introduced by Talebpour and Amoli [23] in 2006. LGP is characterized by reduction of the total gastric volume through a reversible plication of the greater curvature, without the need for a resection or costly stapling devices [24]. A recent meta-analysis by Ye et al. reported that LSG was superior to LGP in terms of weight loss, comorbidities improvement, and postoperative hospital stay [25]. However, further studies compared the two techniques, with conflicting results regarding postoperative efficacy and safety [26–29].

### 1.2. Objectives

As a result, a meta-analysis was performed in order to provide an up-to-date comparison between the two techniques, incorporating also the results of the recent trials, in terms of postoperative complications and reoperation rate, weight loss, cost, and comorbidities improvement.

## 2. Materials and Methods

### 2.1. Study Protocol

The present meta-analysis was conducted based on the principles described in the *Cochrane Handbook for Systematic Reviews of Interventions* and the PRISMA guidelines [30]. The present study was not registered in any database.

### 2.2. Primary Endpoint

As a primary endpoint of this study, was considered the pooled odds ratio (OR) for the overall postoperative complication rate between laparoscopic gastric plication and laparoscopic sleeve gastrectomy procedures in patients who were operated for morbid obesity.

### 2.3. Secondary Endpoints

The secondary endpoints included comparisons in terms of specific (anaemia, abdominal pain, nausea and vomiting, fistula and leak, haemorrhage surgical site infection, invagination, and stenosis) postoperative complications and certain bariatric surgery endpoints, such as % excess weight loss (%EWL), body mass index (BMI), and BMI loss (BMIL) at fixed time points (3, 6, 12, and 36 months postoperatively). Furthermore, pooled comparisons regarding the length of hospital stay (LOS), the operative duration, the reoperation rate, the operation cost, and the postoperative comorbidities (hypertension, diabetes, and sleep apnea) improvement rate were also performed.

### 2.4. Eligibility Criteria

Eligible studies were retrospective or prospective studies, with a morbid obese study population, whose outcomes of interest could be retrieved and were reported in English. More specifically, the included studies should embody in their design algorithm, a comparison between laparoscopic gastric plication and laparoscopic sleeve gastrectomy.

Exclusion criteria for this meta-analysis were as follows: (1) nonhuman studies and trials; (2) not written in English; (3) with no outcome of interest, (4) with no comparison group, or (5) with irretrievable outcome data; and (6) publications in the form of editorials, letters, conference abstracts, and expert opinions.

### 2.5. Literature Search

In order to identify and retrieve the eligible studies, a systematic literature search in electronic scholar databases (MEDLINE, Cochrane Central Register of Controlled Trials, and Scopus and Web of Science) was performed. The last search date was 24 March 2018. The following search Boolean algorithm was applied: plication or imbrication, and sleeve.

### 2.6. Study Selection and Data Collection

The first step of the systematic literature review included the identification and removal of duplicate entries. After that, the titles and abstracts of the studies were screened and sorted on the basis of the above-mentioned eligibility criteria. The final screening process was the full-text review of the remaining trials, in order to assess the consistency with the inclusion key points. The electronic database search, the study selection, the data extraction, and the methodological and quality evaluation of the included studies were all performed in duplicate and blindly by two independent researchers (P. K. and S. E.). In case of a discrepancy, through mutual revision and discussion, a consensus was reached. If the disagreement was not resolved, then the opinion of a third investigator (Z. D.) was considered.

The following data were retrieved from the eligible studies: first author's name, study type, trial location and year, sample size, age and gender of the participants, follow -up duration, preoperative BMI and comorbidities rates, perioperative characteristics, and surgical technique parameters (e.g., previous abdominal surgery, number of trocars, bougie size, pneumoperitoneum level, and number and experience of surgeons), length of hospital stay, operative time, conversion and reoperation rates, operative costs, complications (e.g., anaemia, abdominal pain, nausea and vomiting, fistula and leak, haemorrhage, surgical site infection, invagination, stenosis, and mortality), postoperative %EWL, BMI, BMIL, and comorbidities improvement rate.

All the included studies were submitted to rigorous quality and methodological evaluation. The eligible RCTs underwent assessment on the basis of the Cochrane's Risk of Bias Assessing tool [31]. Rating based on this tool was performed in terms of selection, performance, detection, attrition, and reporting methodology bias. Each endpoint was appointed a color grade, with green and yellow representing low and unclear risk level, while red was regarded as a high risk level. The Newcastle–Ottawa Scale (NOS) was introduced for the assessment of non-RCT studies [32]. The validity checkpoints of this tool, included the selection and comparability of the study groups and the confirmation of the exposure. Each trial was appointed a score ranging from 0 to 9. Cohen's *k* statistic was calculated for both assessment tools.

### 2.7. Statistical Analysis

The Cochrane Collaboration RevMan version 5.3 and IBM SPSS version 23 were utilized for the performance of data analysis and statistical computations. Primary and secondary endpoints were displayed in the form of odds ratio and weighted mean difference (WMD), for dichotomous and continuous variables, respectively. All the results were reported with the corresponding 95% confidence interval (95% CI).

In case that the included trials did not provide the mean or the standard deviation of the continuous variables, they were calculated form the respective median and interquartile range (IR), as described by Hozo et al. [33]. More specifically, if the sample size was >25, then the mean was considered equivalent to the median. SD was estimated as IR/4 and IR/6 for sample sizes <70 and >70, respectively. For dichotomous variables, the statistical method applied, was the Mantel–Haenszel (MH) and for continuous variables, the inverse variance (IV). Both the fixed effects (FE) and the random effects (RE) models were estimated. The model that was finally used was based on the Cochran Q-test. In case of the presence of a statistically significant heterogeneity level (Q-test *p* < 0.1), then the RE model was applied. Quantification of the heterogeneity levels was also performed through the calculation of *I* ^2^. Statistical significance was considered at the level of *p* < 0.05.

### 2.8. Risk of Bias across Studies

The funnel plot of the primary endpoint was visually inspected, in order to determine the presence of publication bias. Based on the primary outcome, an Egger's test was also performed.

## 3. Results

### 3.1. Study Selection

**Figure 1:**
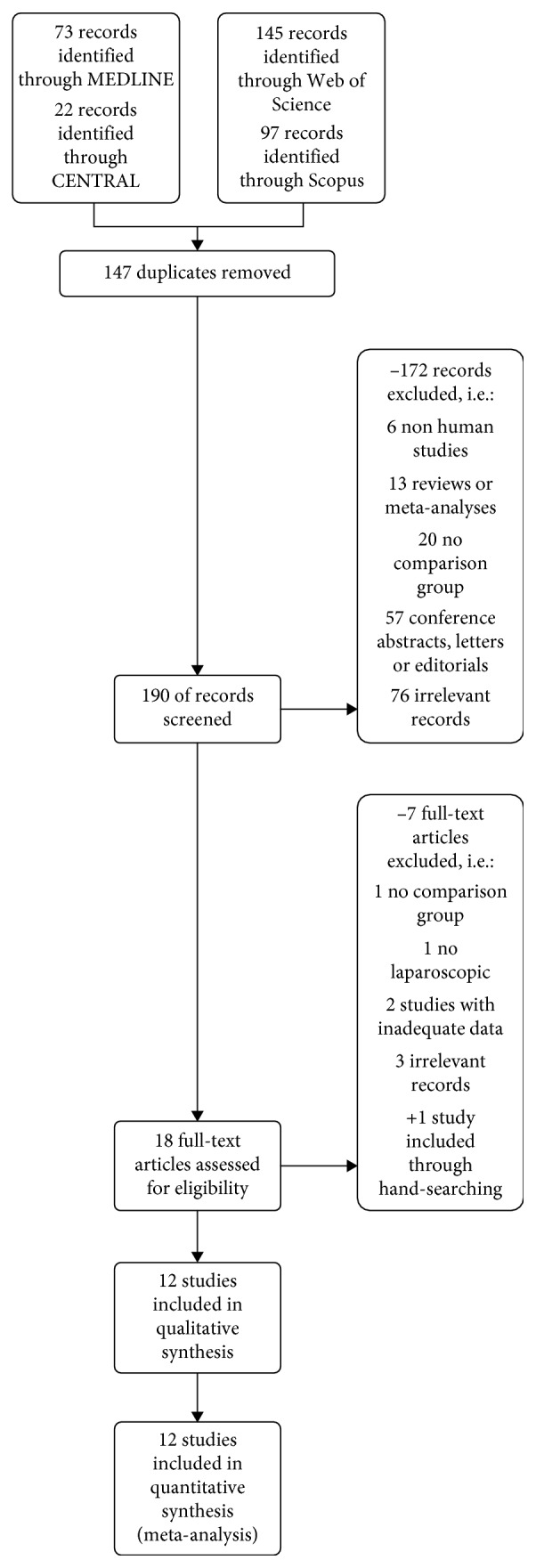
Flow diagram.

Electronic database screening through the application of the above-mentioned algorithm resulted in the extraction of 337 entries (Figure 1). More specifically, the records retrieved were 73, 22, 145, and 97 from MEDLINE, Cochrane Central Register of Controlled Trials, and Web of Science and Scopus, respectively. After the removal of 147 duplicates records, 190 titles and abstracts were screened. In this first phase, 172 studies (6 nonhuman, 13 reviews or meta-analyses, 20 with no comparison group, 57 conference abstracts, letters or editorials, and 76 irrelevant records) were excluded. In the second phase of the literature search, the remaining 18 trials were submitted to a full-text review in order to assess consistency with the predefined eligibility criteria. The full-text screening resulted in the identification and removal of 7 articles (1 with no comparison group, 1 not laparoscopic, 2 studies with inadequate data, and 3 irrelevant records). Furthermore, through hand searching of the current bibliography, 1 study was introduced. Consequently, 12 trials [26–29, 34–41] were included in the qualitative and quantitative synthesis of the present meta-analysis.

### 3.2. Study Characteristics

The characteristics of the eligible studies are summarized in Table 1. As far as the study type was concerned, 4 trials [29, 36, 37, 40] had a RCT design, while 3 studies [27, 28, 34] reported a prospective and 5 studies [26, 35, 38, 39, 41], a retrospective layout, respectively. In total, 7 trials [26, 29, 34–36, 38, 39] were conducted in a single institution, and 5 studies [27, 28, 37, 40, 41] incorporated multiple surgical centres. The study completion year spanned from 2013 to 2017. The total sample size was 950 patients. Moreover, Table 1 displays the gender, age, and BMI allocation between the two study groups. Postoperative follow-up extended from 6 months up to 3 years.

Furthermore, 4 studies [27, 28, 35, 39] did not provide any data regarding coexisting comorbidities (Table 2). More specifically, data extraction from the eligible studies resulted in the identification of 114, 74, and 33 obese patients presenting with hypertension, diabetes mellitus, and sleep apnea, respectively. In total, 32 patients had been submitted to abdominal operations prior to the studied obesity surgery. The majority of LSG and LGP procedures were performed with 5 trocars [27, 34, 35, 38–40] although techniques using 4 [36, 37, 41] or even 3 [41] trocars were reported. Although 2 studies [28, 41] did not provide information regarding the intraoperative use of a bougie, the recorded bougie size ranged from 32 Fr to 42 Fr. It must be noted, though, that in the study by Bužga et al. [27], no bougie was applied during the sleeve gastrectomy or the gastric plication. Similarly, the pneumoperitoneum gas pressure extended from 12 mmHg to 15 mmHg. The operations were performed either by a surgical team [29, 40] or a single surgeon [26, 35, 38, 39, 41]. Operative experience of the involved surgeons was validated in only two trials [35, 38].

### 3.3. Risk of Bias within Studies

Table 3 displays a summary of the methodology and quality evaluation of the non-RCT eligible studies, on the basis of the Newcastle–Ottawa Scale. The quality level of the included studies was estimated to be in adequate level, since the overall score ranged between 5 and 8 stars. Furthermore, Table 4 represents a brief report of the results of the Cochrane's Risk of Bias Assessing tool for the included RCTs. Low risk of bias was identified in the fields of random sequence generation, incomplete outcome bias, and selective reporting. Concerning the interrater agreement between the two investigators, it was estimated to be in very good levels, in both tools (NOS Cohen's *k* statistic: 86%, *p* < 0.001 and Cochrane's Risk of Bias Assessing tool Cohen's *k* statistic: 76.3%, *p* < 0.001), thus confirming the absence of any major discrepancy during the evaluation process.

### 3.4. Primary Endpoint

**Figure 2:**
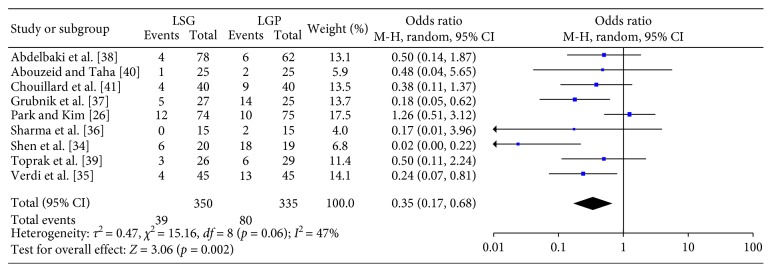
Overall complications.

As far as the primary endpoint is concerned, 9 studies provided data for the comparison between LSG and LGP in terms of overall complications (Figure 2). Meta-analysis of these data showed a statistically significant (*p*=0.002) lower rate of complications in favor of the LSG group (OR: 0.35; 95% CI: 0.17, 0.68). Heterogeneity between the studies was significant (Q-test *p*=0.06, *I* ^2^=47%), and as a result, a RE model was applied.

Due to the fact that high levels of heterogeneity were observed, further analysis was performed.  Figure 11, displays a summary of the sensitivity analysis of the included trials. Meta-regression in terms of sample size (*p*=0.078), follow-up (*p*=0.509), and bougie size (*p*=0.294) did not yield statistically significant results. Moreover, subgroup analysis was performed, in order to investigate other possibly heterogeneity introducing factors. Heterogeneity was reduced (Q-test *p*=0.17, *I* ^2^=38%), without deviation from the overall result (OR: 0.26; 95% CI: 0.13, 0.49; *p* < 0.0001), in the analysis of the studies reporting the 5 trocar techniques. Similarly, the presence of a single (OR: 0.56; 95% CI: 0.33, 0.93; *p*=0.03) or an experienced (OR: 0.33; 95% CI: 0.14, 0.80; *p*=0.01) surgeon for the conduction of the LSG and LGP operations significantly influences the levels of heterogeneity (Q-test *p*=0.26, *I* ^2^=25%; and Q-test *p*=0.41, *I* ^2^=0%, respectively). Further analysis, in terms of other factors, was not performed due to the inconsistency and scarcity of the provided data.

### 3.5. Secondary Endpoints

**Figure 3:**
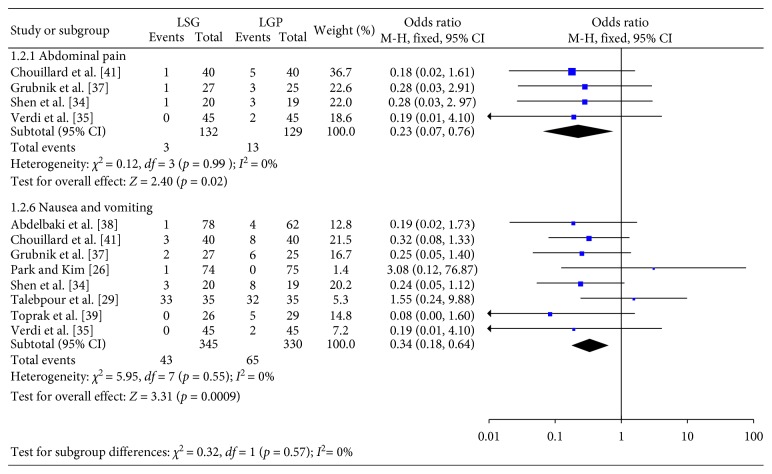
Minor complications.

Figure 3 summarizes the data regarding the comparisons between the two groups, in terms of minor complications. Statistically significant lower rates of abdominal pain (OR: 0.23; 95% CI: 0.07, 0.76; *p*=0.02) and nausea and vomiting (OR: 0.34; 95% CI: 0.18, 0.64; *p*=0.0009) in the LSG group were recorded. Since significant heterogeneity was not confirmed (Q-test *p* > 0.1 in all comparisons), a FE model was used.

**Figure 4:**
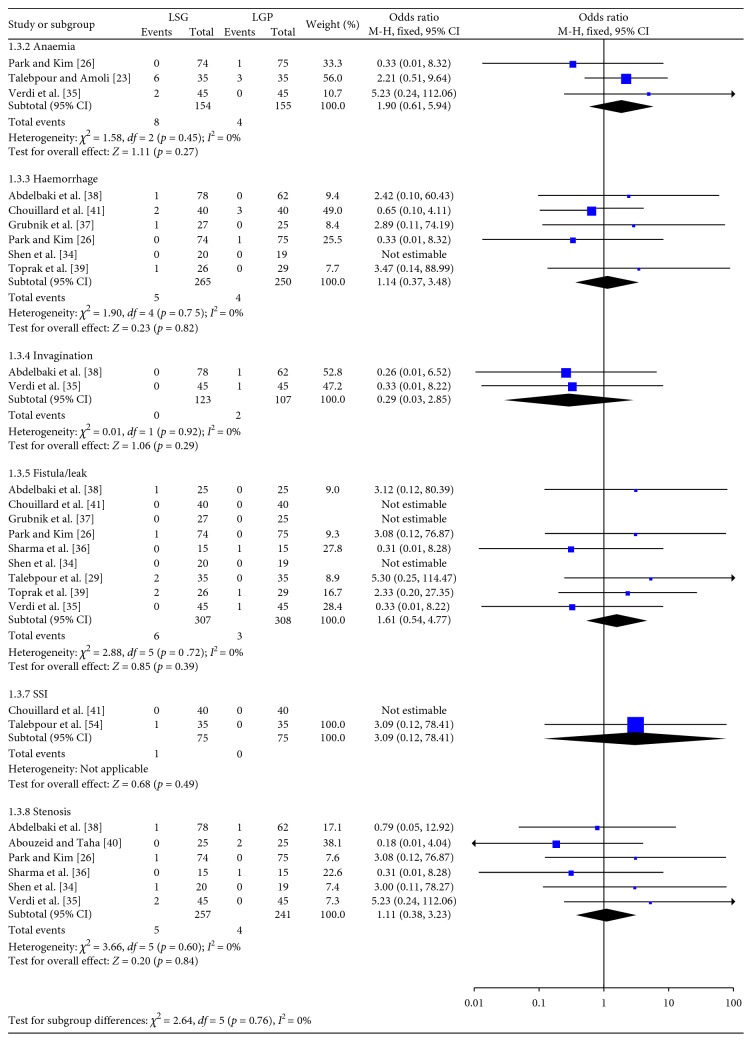
Major complications.

In Figure 4, the pooled results of the eligible studies concerning the major complications are depicted. Analysis of the extracted data showed no significant difference in the rates of anaemia (OR: 1.90; 95% CI: 0.61, 5.94; *p*=0.27), haemorrhage (OR: 1.14; 95% CI: 0.37, 3.48; *p*=0.82), invagination (OR: 0.29; 95% CI: 0.03, 2.85; *p*=0.29), fistula or leak (OR: 1.61; 95% CI: 0.54, 4.77; *p*=0.39), SSI (OR: 3.09; 95% CI: 0.12, 78.41; *p*=0.49), and stenosis (OR: 1.11; 95% CI: 0.38, 3.23; *p*=0.84). Heterogeneity between the studies was not significant (Q-test *p* > 0.1 in all comparisons), and as a result, a FE model was applied.

**Figure 5:**
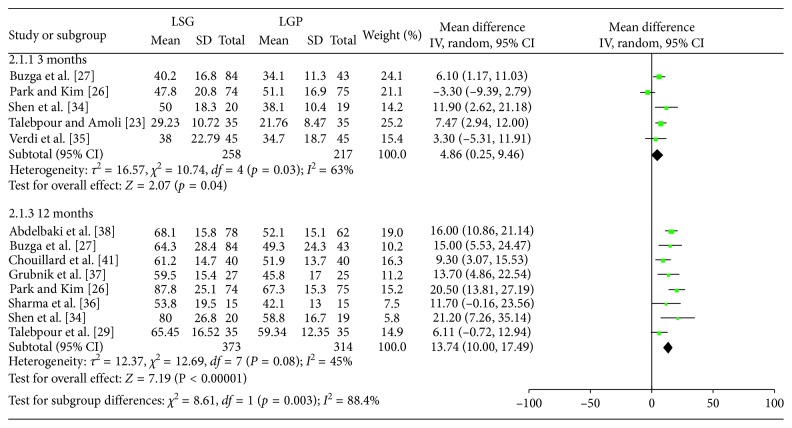
%EWL at 3 and 12 months.

**Figure 6 fig6:**
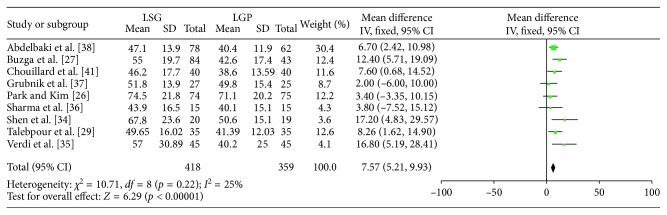
%EWL at 6 months.

**Figure 7:**
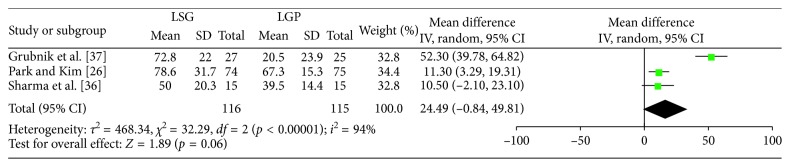
%EWL at 36 months.

Figures 5 [Fig fig6]–7 summarize the data regarding the comparisons between the two groups, in terms of %EWL. More specifically, statistically significant higher %EWL in the LSG group was identified at 3 (WMD: 4.86; 95% CI: 0.25, 9.46; *p*=0.04), 6 (WMD: 7.57; 95% CI: 5.21, 9.93; *p* < 0.00001), and 12 (WMD: 13.74; 95% CI: 10, 17.49; *p* < 0.0001) months, postoperatively. No significant result was identified at 36 months (WMD: 24.49; 95% CI: −0.84, 49.81; *p*=0.06). Heterogeneity between the studies was significant at 3, 12, and 36 months (Q-test *p*=0.03, *I* ^2^=63%; Q-test *p*=0.08, *I* ^2^=45% and Q test *p* < 0.00001, *I* ^2^=94%, respectively) and as a result, a RE model was applied. On the contrary, a FE model (Q test *p*=0.22, *I* ^2^=25%) was applied at 6 months.

Figures 3.1 and 3.2 (Supplementary Material) summarize the data regarding the comparisons between the two groups, in terms of BMI. More specifically, statistically significant higher BMI was identified in the LGP group at 6 (WMD: −0.88; 95% CI: −1.63, −0.13; *p*=0.02) and 12 (WMD: −1.19; 95% CI: −1.97, −0.41; *p*=0.003) months, but not at 3 (WMD: 1.04; 95% CI: −1.69, 3.78; *p*=0.45) months. Heterogeneity between the studies was not significant at 6 and 12 months (Q-test *p*=0.02, *I* ^2^=37%; Q-test *p*=0.55, *I* ^2^=0%), and as a result, a FE model was applied. On the contrary, a RE model (Q-test *p*=0.004, *I* ^2^=82%) was applied at 3 months.

Figure 4.1 (Supplementary Material) summarizes the data regarding the comparisons between the two groups, in terms of BMIL. More specifically, statistically significant higher BMIL in the LSG group was identified at 12 months (WMD: 2.19; 95% CI: 1.28, 3.10; *p* < 0.00001), but not at 6 (WMD: 1.18; 95% CI: −0.88, 3.23; *p*=0.26) months. Since heterogeneity was significant at 6 months (Q -test *p*=0.02, *I* ^2^=82%) and the overall number of the included studies was small, a RE model was applied.

Figure 5.1 (Supplementary Material) summarizes the data regarding the comparison between the two groups, in terms of LOS. More specifically, no statistically significant difference (WMD: 0.49; 95% CI: −0.19, 1.17; *p*=0.16) between the two groups, regarding LOS, was identified. Since heterogeneity was significant (Q-test *p* < 0.00001, *I* ^2^=93%), a RE model was applied.

Figure 6.1 (Supplementary Material) summarizes the data regarding the comparison between the two groups, in terms of operative time. More specifically, no statistically significant difference (WMD: 1.27; 95% CI: −9.00, 11.53; *p*=0.81) between the two groups, regarding operative time, was identified. Since heterogeneity was significant (Q test *p* < 0.00001, *I* ^2^=91%), a RE model was applied.

Figure 7.1 (Supplementary Material) summarizes the data regarding the comparison between the two groups, in terms of reoperation rate. More specifically, no statistically significant difference (OR: 0.59; 95% CI: 0.13, 2.78; *p*=0.51) between the two groups, regarding reoperation rate, was identified. Since heterogeneity was significant (Q-test *p*=0.01, *I* ^2^=65%), a RE model was applied.

Figure 8.1 (Supplementary Material) summarizes the data regarding the comparison between the two groups, in terms of cost. More specifically, no statistically significant difference (WMD: 2921.07; 95% CI: −107.07, 5949.21; *p*=0.06) between the two groups, regarding cost, was identified. Since heterogeneity was significant (Q-test *p* < 0.00001, *I* ^2^=10%), a RE model was applied.

Figure 9.1 (Supplementary Material) summarizes the data regarding the comparisons between the two groups, in terms of comorbidities improvement. More specifically, no statistically significant difference between the two groups, regarding improvement in hypertension (OR: 0.66; 95% CI: 0.25, 1.75; *p*=0.41), diabetes (OR: 1.05; 95% CI: 0.18, 6.1; *p*=0.96), and sleep apnea (OR: 0.35; 95% CI: 0.05, 2.63; *p*=0.31), was identified. Since heterogeneity was not significant (Q-test *p*=0.34, *I* ^2^=12%; Q-test *p*=0.83, *I* ^2^=0%; and Q-test *p*=0.32, *I* ^2^=0%, respectively), a RE model was applied.

### 3.6. Risk of Bias across Studies

Figure 10 (Supplementary Material) displays the funnel plot of the primary outcome. Through visual inspection, a symmetrical distribution of the included studies was confirmed. Similarly, Egger's test was not statistically significant, thus excluding the presence of publication bias (*p*=0.105).

## 4. Discussion

### 4.1. Summary of Evidence

The bariatric surgery quality improvement program, through a recognized accreditation process and a platform-based clinically derived data registry, resulted in the reduction of operative costs and the decrease of the complication rates [42, 43]. As far as the overall complication rate was concerned, it decreased from 4.6% in 2006 to 3% in 2013 [42]. Despite the minor, early complications such as nausea, vomiting, and abdominal pain, severe adverse events (haemorrhage, leak, etc.) have a devastating impact on the postoperative course and may even require operative management. The incidence rate of these complications can reach up to 3%, with a correlated mortality rate equal to 0.2% [44, 45]. More specifically, regarding LSG, the overall postdischarge morbidity and serious morbidity rates were estimated at 2.48% and 0.89%, respectively [42]. Furthermore, the frequency of perioperative leak was 3.93%, and the respective rate of bleeding was 4.07% [46]. Staple line leak is described as one of the most feared post-LSG complications, due to the derived morbidity and the frequent requirement for specialized treatment, such as OTSC and stents [19, 47]. Gastric plication or gastric imbrication has been recently introduced and proposed as an alternative to LSG. LGP is characterized similar to LSG, but reversible, gastric tube formation and elimination of the greater curvature without the need for gastrectomy or staple lines [48]. Despite the absence of anastomotic lines and the subsequent risk for leaks, fistulas and haemorrhage, the double row stitching, and the large stomach folds imbricated in the gastric lumen result in increased rate of nausea and vomiting [49]. Summing up the results from all trials comparing LSG and LGP, we demonstrated that, in total, LSG demonstrates a significantly safer postoperative profile, when compared to LGP. More specifically, when categorized, LGP was associated with statistically significant higher rates of benign minor postoperative complications, such as abdominal pain, nausea, and vomiting. Regarding severe complications (i.e., haemorrhage, fistula, leak, SSI, etc.) that are in fact the main factors that affect postoperative outcome, the rates were comparable between the two techniques.

Postoperative complications are considered as a distinct factor that affects length of hospital stay. However, recent trials have questioned this statement concerning hospitalization after bariatric procedures [50]. According to a retrospective analysis of 2629 patients submitted to LSG by Jakob et al. [51], hospital stay beyond 24 hours is not obligatory, under the condition that there are no signs of bleeding and leak or symptoms of vomiting and nausea. The reasoning under these conclusions was that major complications occur within the first 24 hours or after the fifth postoperative day. Similarly, LGP has been proposed by Waldrep and Pacheco [52] as a safe and effective bariatric procedure that can be performed in an outpatient basis, since there is no need for gastric resection, anastomosis, or foreign bodies. The present systematic review revealed that the two techniques were comparable in terms of LOS and that mean LOS ranged from 1.9 to 7.46 days in the LSG group and from 1.2 to 6.06 days in the LGP group.

Weight loss is the commonest outcome, by which the efficacy of a bariatric procedure is estimated. Based on animal studies, gastric plication and sleeve gastrectomy displayed the same efficacy, concerning weight loss, reduced food intake fasting plasma glucose and intraperitoneal glucose tolerance test, and both procedures affected ghrelin and GLP-1 levels [53]. Although gastric plication has functional restrictive effects [54], sleeve gastrectomy affects residual gastric volume, gastrointestinal tract motility, and hormonal balance [55]. The competence of gastric plication in weight loss has been demonstrated in clinical settings [56, 57]. Comparative studies for LSG and LGP are not conclusive [26, 29, 35]. Meta-analysis of these trials confirmed the short-term and medium-term superiority of LSG in %EWL. However, there was no difference at long-term results (i.e., 3 years postoperatively). This analysis, however, suffered from great heterogeneity levels and included only a few studies. Mean BMI was higher in the LGP group at 6 and 12 months postoperatively, and LSG portrayed a higher medium-term BMIL. Long-term analysis of BMI and BMIL was impossible due to scarcity of data. Furthermore, in contrast to current literature reports [58], we did not identify any difference regarding reoperation rates.

Despite the fact that LSG and LGP do not involve an extensive rerouting of the gastrointestinal tract that is performed in other operations, such as RYGB, and since they incorporate the minimal invasive principles, they are both considered as technically demanding. According to a study from our institution [59], the learning curve, concerning laparoscopic sleeve gastrectomy in a newly established bariatric centre, stabilized at 68 procedures. Due to these facts and taking into consideration the different technical aspects between the two techniques (e.g., use of linear endostaplers versus application of extramucosal stitches), a difference in the operation duration would be justified. The pooled analysis in our study did not identify any discrepancy, though, in the time needed for LSG or LGP to be performed.

Besides these, the operative charges for bariatric surgery are another subject of extensive research, since the ideal operation should be characterized by optimum excess weight loss, minimization of complications rate, and reduced economic cost. Based on recent studies, the mean expenditures for the performance of LSG are not negligible [60]. Technical characteristics, such as the use of staplers also contribute to the overall increased cost [61, 62]. The introduction of LGP as an alternative restrictive procedure, where no staplers, but only stitches are used, aimed at achieving an equivalent bariatric result at nonabundant economic settings. Primary results reported the inferior cost of an LGP procedure, when compared to LSG [34, 41]. However, our pooled results did not confirm these findings, since no statistically significant difference was found. The increased heterogeneity, the minimum study sample, and the fact that, in one study, [34] the total operative cost was reported, while in the other trial [41], the mean operating room technical cost was displayed, confining the significance of the analysis.

Obesity is a renowned predisposing factor for various comorbidities. Characteristically, according to the recent study by Pantalone et al. [63], the prevalence of type II diabetes and prediabetes increased from 4.5% and 0.9% in the BMI < 25 group to 30.9% and 16.9% in the BMI > 40 group, respectively. In a meta-analysis by Guh et al. [64], the pooled IRR estimate for hypertension in obese males was 1.84 (95% CI: 1.51, 2.24) and in females 1.90 (95% CI: 1.77, 2.03). Moreover, the obstructive sleep apnea rates in obese patients are increased, ranging from 21% to 43% [60]. Various trials have compared the efficacy of LSG and LGP in terms of comorbidities improvement rates, without identifying any significant difference [26, 34, 38]. Similarly, our meta-analysis estimated that LSG and LGP are equivalent in terms of hypertension, diabetes, and sleep apnea improvement.

### 4.2. Limitations

Before taking into account the outcomes reported in our study, careful consideration of specific study limitations should be performed. Firstly, despite the fact that quality and methodological evaluation of the eligible studies generated satisfactory results, nonhomogeneity in the study type could possibly introduce a certain amount of bias. More specifically, the lack of randomization and blinding in the introduced prospective or retrospective studies, possibly contributes in the recorded heterogeneity levels. Furthermore, since the trial sample size in both comparison groups was small, the significance of the derived meta-analysis results was compromised. Moreover, as validated in the subgroup analysis, the inconsistency in the reported number and experience of the operating surgeons constitute an important bias introducing factor. Diversity was substantial in certain reported technical characteristics, such as the number of trocars used, the boogie size, the pneumoperitoneum pressure levels, and the volume reduction technique. Subanalysis, confirmed the influence of the number of trocars on the overall heterogeneity. Despite that, the existence of variations in the rest of the above-mentioned technical key points reduces the credibility of the meta-analysis outcomes, through introduction of bias. Similarly, although a correlation between follow-up duration and the primary endpoint was not established, certain amount of bias should be anticipated due to the inconsistency in the length of follow-up. Finally, the divergence in preoperative comorbidities, mean BMI levels, and the absence of systematic obesity classification further inhibits the effort for homogeneous and consistent pooled outcomes.

## 5. Conclusions

The present meta-analysis represents an attempt to provide an up-to-date and in-depth evaluation of laparoscopic gastric plication and laparoscopic sleeve gastrectomy in morbid obesity. Supremacy of laparoscopic sleeve gastrectomy in terms of overall complications rate, minor postoperative complications, and short- and medium-term weight loss was documented. However, no statistically significant difference between the two operative techniques regarding the major postoperative complications, the length of hospital stay, the operation duration, the reoperation rate, or the cost was found. Taking into consideration the above-mentioned results and several study limitations, we can safely claim that evidence exists for the superiority of LSG over LGP in terms of overall complications rate and postoperative weight loss. However further prospective randomized trials, with a higher methodological quality level, are needed in order to validate these results.

## Figures and Tables

**Table 1:** Study characteristics.

ID	Author	Country	Type of study	Centre	Year	Group	Sample	Gender (M/F)	Age	BMI	Follow-up
25066442	Abdelbaki et al. [38]	Egypt	Retrospective	Single centre	2015	LSG	78	21 (27%)/57 (73%)	31.77 (9.2)	48.27 (6.9)	12 months
						LGP	62	12 (19%)/50 (81%)	34.45 (10.7)	41.62 (7.1)	

0.4103/1110-1121.153370	Abouzeid and Taha [40]	Egypt	RCT	Multicentre	2015	LSG	25	7 (28%)/18 (72%)	34.8 (11.3)	46.8 (3.7)	24 months
						LGP	25	9 (36%)/16 (64%)	32.1 (8.8)	47.8 (3.8)	

28674838	Bužga et al. [27]	Czech Republic	Prospective	Multicentre	2017	LSG	84	23 (27.3%)/61 (72.6%)	42 (10.3)	43.7 (5.4)	18 months
						LGP	43	15 (34.8%)/28 (65.1%)	42.5 (8)	42.5 (5.5)	

10.1089/bari.2016.0022	Bužgová et al. [28]	Czech Republic	Prospective	Multicentre	2016	LSG	43	13 (30.2%)/30 (69.8%)	44.5 (10.5)	42.8 (5.4)	12 months
						LGP	25	10 (40%)/15 (60%)	43.5 (8.1)	42.2 (5.6)	

26482166	Chouillard et al. [41]	France	Retrospective	Multicentre	2015	LSG	40	4 (10%)/36 (90%)	35.4 (6.65)	41.2 (5.17)	18 months
						LGP	40	4 (10%)/36 (90%)	34.2 (8.15)	40.4 (4.01)	

14322218	Grubnik et al. [37]	Ukraine	RCT	Multicentre	2015	LSG	27	7 (25.9%)/20 (74.1%)	44.2 (6.8)	45.8 (7.2)	3 years
						LGP	25	5 (20%)/20 (80%)	40.5 (5.2)	41.6 (6.5)	

28792149	Park and Kim [26]	Korea	Retrospective	Single centre	2017	LSG	74	16 (21.6%)/58 (78.4%)	30.4 (7.9)	34.7 (53.6)	3 years
						LGP	75	8 (10.7%)/67 (89.3%)	32.6 (6.7)	33.7 (3.3)	

25428511	Sharma et al. [36]	India	RCT	Single centre	2014	LSG	15	9 (60%)/6 (40%)	39.9	44.0 (7.8)	3 years
						LGP	15	9 (60%)/6 (40%)	40.5	44.7 (6.1)	

23443480	Shen et al. [34]	China	Prospective	Single centre	2013	LSG	20	7 (35%)/13 (65%)	34.2 (6.3)	38.4 (6.3)	12 months
						LGP	19	5 (26.3%)/14 (73.7%)	33.9 (5.7)	37.3 (4.3)	

29043548	Talebpour et al. [29]	Iran	RCT	Single centre	2017	LSG	35	6 (17.1%)/29 (82.9%)	38.6 (10.27)	44.6(3.5)	24 months
						LGP	35	8 (22.9%)/27 (77.1%)	35.34 (10.08)	48.39(4.89)	

26985155	Toprak et al. [39]	Turkey	Retrospective	Single centre	2015	LSG	26	21 (80.8%)/5 (19.2%)	33.9 (10.4)	42.0 (3.1)	12 months
						LGP	29	23 (79.3%)/6 (20.7%)	35.5 (11.2)	41.4 (3.0)	

25663148	Verdi et al. [35]	Italy	Retrospective	Single centre	2015	LSG	45	6 (13.3%)/39 (86.7%)	40 (9.14)	41(5.07)	6 months
						LGP	45	6 (13.3%)/39 (86.7%)	37.8 (11.45)	40.65(4.99)	

**Table 2:** Comorbidities and surgical technique.

Author	Group	Comorbidities			Previous operations	Technique					
		Hypertension	Diabetes	Sleep apnea		Trocars	Boogie	Pneumoperitoneum	Volume reduction	Surgeons	Experience
Abdelbaki et al. [38]	LSG	12	7	n/a	0	5	32 Fr	14-15 mmHg	Linear endostapler	1	Yes
	LGP	10	2		0	5	32 Fr	14-15 mmHg	2 rows of extamucosal stiches	1	Yes

Abouzeid and Taha [40]	LSG	8	n/a	n/a	n/a	5	36 Fr	n/a	Linear endostapler	Surgical team	n/a
	LGP	4				5	36 Fr		2 rows of extamucosal stiches		

Bužga et al. [27]	LSG	n/a	n/a	n/a	n/a	n/a	No boogie	n/a	Linear endostapler	n/a	n/a
	LGP					5	No boogie		2 rows of extamucosal stiches		

Bužgová et al. [28]	LSG	n/a	n/a	n/a	n/a	n/a	n/a	n/a	n/a	n/a	n/a
	LGP										

Chouillard et al. [41]	LSG	8	7	3	8	3 or 4	40 Fr	n/a	Linear endostapler	1	n/a
	LGP	9	5	4	6	3 or 4	40 Fr		2 rows of extamucosal stiches	1	

Grubnik et al. [37]	LSG	7	3	4	0	4	32 Fr	12–14 mmHg	Linear endostapler	n/a	n/a
	LGP	5	2	4	0	4	32 Fr	12–14 mmHg	2 rows of extamucosal stiches		

Park and Kim [26]	LSG	15	11	12	n/a	n/a	36 or 40 Fr	n/a	Linear endostapler	1	n/a
	LGP	7	5	4			36 Fr		2 rows of extamucosal stiches	1	

Sharma et al. [36]	LSG	0	8	n/a	n/a	4	40 Fr	n/a	Linear endostapler	n/a	n/a
	LGP	6	3			4	40 Fr		2 rows of extamucosal stiches		

Shen et al. [34]	LSG	4	3	1	1	5	32 Fr	12–14 mmHg	Linear endostapler	n/a	n/a
	LGP	3	2	1	0	5	32 Fr	12–14 mmHg	2 rows of extamucosal stiches		

Talebpour et al. [29]	LSG	9	10	n/a	n/a	n/a	n/a	n/a	Linear endostapler	Surgical team	n/a
	LGP	7	6						2 rows of extamucosal stiches		

Toprak et al. [39]	LSG	n/a			n/a	5	42 Fr	12 mmHg	Linear endostapler	1	n/a
	LGP					5	42 Fr	12 mmHg	Extramucosal stiches	1	

Verdi et al. [35]	LSG	n/a			17	5	34 Fr	n/a	Linear endostapler	1	Yes
	LGP				0	5	34 Fr		2 rows of extamucosal stiches	1	Yes

**Table 3:** Newcastle–Ottawa Scale.

Study	Selection				Comparability	Exposure/Outcome			Total
	1	2	3	4	5	6	7	8	
Abdelbaki et al. [38]		^*∗*^	^*∗*^	^*∗*^			^*∗*^	^*∗*^	5

Bužga et al. [27]		^*∗*^	^*∗*^	^*∗*^		^*∗*^	^*∗*^	^*∗*^	6

Bužgová et al. [28]		^*∗*^	^*∗*^	^*∗*^		^*∗*^	^*∗*^	^*∗*^	6

Chouillard et al. [41]	^*∗*^		^*∗*^	^*∗*^	^*∗∗*^	^*∗*^	^*∗*^	^*∗*^	8

Park and Kim [26]	^*∗*^	^*∗*^				^*∗*^	^*∗*^	^*∗*^	5

Shen et al. [34]		^*∗*^	^*∗*^	^*∗*^			^*∗*^	^*∗*^	5

Toprak et al. [39]	^*∗*^			^*∗*^		^*∗*^	^*∗*^	^*∗*^	5

Verdi et al. [35]	^*∗*^			^*∗*^		^*∗*^	^*∗*^	^*∗*^	5

**Table 4:** Cochrane's Risk of Bias Assessing Tool.

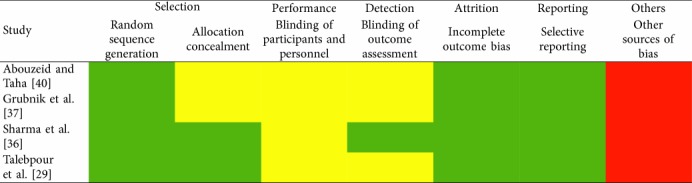

## Supplementary Materials

Supplementary Materials Figure 3.1: BMI at 3 months; Figure 3.2: BMI at 6 and 12 months; Figure 4.1: BMIL; Figure 5.1: LOS; Figure 6.1: operation duration; Figure 7.1: reoperation; Figure 8.1: cost; Figure 9.1: comorbidities improvement; Figure 10: funnel plot; Figure 11: sensitivity analysis.
